# Environmental risk assessment of priority biocidal substances on Polish surface water sample

**DOI:** 10.1007/s11356-020-11581-7

**Published:** 2020-11-21

**Authors:** Justyna Durak, Tomasz Rokoszak, Alicja Skiba, Przemysław Furman, Katarzyna Styszko

**Affiliations:** 1 Faculty of Energy and Fuels, Department of Coal Chemistry and Environmental Sciences, AGH University of Science and Technology, Krakow, Poland; 2 Faculty of Physics and Applied Computer Science, Department of Applied Nuclear Physics, AGH University of Science and Technology, Krakow, Poland

**Keywords:** Biocides, Surface water, Water pollution, Ecological risk assessment (ERA), Toxicity, Gas chromatography-mass spectrometry

## Abstract

The EU directive 2013/39/EU has incorporated four biocidal compounds as priority substances: diuron, isoproturon, cybutryne, and terbutryn. The research was undertaken to determine the concentration of biocides in surface waters in three locations in southern Poland: the Wisła River in Kraków, the Wisłoka River in Mielec, and the drainage ditch draining water from arable fields located near Mielec. Environmental samples were taken in two series: winter (February) and spring (May and June). The analyses were carried out using gas chromatography with mass spectrometry. The seasonality of biocides in surface waters was observed. In winter samples, the concentrations were below MQL, while in spring, they ranged from a few to several dozen nanograms per liter. The highest concentrations of all analyzed compounds were recorded in water taken from the Wisła River. According to directive 2013/39/EU, the maximum allowable concentration was exceeded only in the case of cybutryne in water from the Wisła, both in May and in June. The assessment of the toxicity with the tested compounds was defined based on the Environmental Risk Assessment method. Low risk was estimated for diuron and isoproturon, while moderate risk for terbutryn and cybutryne.

## Introduction

The rapid development of industry means that the natural environment is exposed to all kinds of contamination with toxic organic and inorganic compounds. This problem concerns especially soils and waters, both surface and underground. One of the most serious threats to the environment is the contamination of surface waters by compounds called biocides (Ormad et al. 2008; Palma et al. 2014; Ccanccapa et al. 2016; Styszko and Kupiec 2016). Biocides are both compounds of natural origin (extracts of herbs), as well as compounds obtained through chemical synthesis (pesticides, seed treatment). These compounds affect the elimination and neutralization of a given group of organisms from the environment, i.e., plants, insects, or fungi. Biocides are a very general term for all substances that enable a fight against harmful organisms (Biziuk 2001). Biocidal preparations are commonly used in the industry and households to prevent spreading organisms, which can be dangerous for human and animal health, as well as organisms which can destroy everyday items (both natural and manufactured) (Kresmann et al. 2018). According to *the EU Regulation of concerning the making available on the market and use of biocidal products*, biocidal products include not only a wide variety of preparations, e.g., hand, surface, and water disinfectants, but also building materials and wood preservatives, rodents, and insect repellents or anti-fouling products (The European Parlament and the Council of the European Union 2012). Biocides are used primarily for the following purposes: to prevent the growth of algae and fungi on building elements such as façades or roofs, or as anti-fouling products applicable to the protective coatings on objects used in the aquatic environment, and in agriculture to prevent spreading organisms with harmful crops (Quednow and Püttmann 2007; Schoknecht et al. 2009; Styszko and Kupiec 2018). These harmful substances enter the water environment during precipitation as a result of their leaching from building materials as well as by being carried with water flowing from the agricultural areas (Bester et al. 2014; Malaj et al. 2014).

Taking into account the diversity of chemical structure, application, toxicity, and persistence in the environment, many classifications of biocides may be indicated. One of the best known classifications of biocides is that which presents four main groups of applications: disinfectants and general biocidal products, preservatives, biocidal products for pest control, and other biocidal products (Ordinance of the Minister of Health 2003). The most numerous subgroups of states include biocides used for conservation purposes.

It was proved that chemical pollutants can cause acute toxicity to aquatic organisms, and accumulation of toxic substances in the ecosystem, which can lead to the loss of habitat and biodiversity, as well as a threat to human health (Mohr et al. 2008; Bollmann et al. 2014a; Styszko et al. 2014). In response to environmental problems, many environmental restrictions and directives regulating pollution-related problems have appeared (Bollmann et al. 2014b). The current directive in the field of water policy is the directive of the European Parliament and of the Council, 2013/39/EU of August 12, 2013, which replaced Directives 2000/60/EC and 2008/105/EC for priority substances in the field of water policy, introducing fifteen new priority substances in reference to various applications: substances contained in plant protection products, industrial chemicals, by-products of combustion, and substances contained in biocidal products. In the last group, four biocides are included the following: isoproturon, diuron, terbutryn, and cybutryne (The European Parlament and the Council of the European Union 2013). Isoproturon is a selective herbicide which belongs to the phenylurea class. This substance fights mono- and dicotyledonous plants. It is a component of preparations used mainly in the protection of cereals (Del Pilar et al. 2001; Bobu et al. 2005). Diuron is a substituted herbicide based on derivatives of urea. Due to its properties, diuron is also present in preparations which are used to prevent spreading algae as well as in coatings as an anti-fouling additive (Armenta et al. 2005). Cybutryne, also called Irgarol, is a strong s-triazine herbicide. It is widely used as a support biocide in anti-fouling paints used, among others, for the maintenance of ship hulls, or in construction to protect the exterior of buildings (Fernandez and Gardinali 2016; Wang et al. 2016; Zhang et al. 2019). Terbutryn is a selective herbicide used to protect cereals, legumes, and fruit trees. Like cybutryne, it also acts as an agent against underwater and floating vegetation (Velisek et al. 2010; Wang et al. 2016). Terbutryn might also be emitted to surface waters from wastewater treatment plants (Quednow and Püttmann 2007; Bollmann et al. 2014a). Occurrence and fate of chemicals, including biocides, in the environment are determined by physical-chemical properties of the compound, environmental conditions, and anthropogenic factors. Among the physical and chemical properties, partition constants, solubilities, or kinetic constants determine biotic and non-biotic mediated reactions. Mobility of chemicals, their availability to participate in leaching, and degradation processes are determined by sorption processes (Rabølle and Spliid 2000; Guillén et al. 2012). Moreover, environmental external factors such as climate, landscape, and biota have definite influence on fate of chemicals. One of the major input information related to the level of concentrations of chemicals in the environment is human activity, determined by consumption of chemicals, mode of use chemical emission rate, and its characteristics.

Table 1 provides information drawn from the current directive on the permissible concentrations tested of biocides. Also, Polish legislation increases the emphasis on ecological requirements as far as the environmental impact of biocidal products is concerned. The abovementioned biocides have been included in the list of priority substances, so their elimination should be a priority in the water protection policy (Ordinance of the Minister of the Environment 2013).

**Table 1:** Biocide concentrations according to the directive 2013/39/UE

The name of the biocide	Concentration of the substance in surface waters Inland (annual intake) (μg/L)	Concentration of the substance in other surface waters (annual intake) (μg/L)	Concentration of the substance in surface waters Inland (maximum permissible) (μg/L)	Concentration of the substance in other surface waters (maximum allowable) (μg/L)
Diuron	0.2	0.2	1.8	1.8
Isoproturon	0.3	0.3	1.0	1.0
Cybutryne	0.0025	0.0025	0.016	0.016
Terbutryn	0.065	0.0065	0.34	0.034

The aim of the work is to present the detection method of selected biocides from the aquatic environment by means of gas chromatography with mass spectrometry and to present the results of the analysis of river waters taken from three locations in the southern part of Poland. These compounds have been covered by water monitoring, like other priority substances. They have not been monitored before; therefore, it is advisable to carry out the relevant studies. The research was directly based on the analysis of biocide concentrations in surface water, due to the WFD focuses on water samples. The level of the toxic risk caused by biocides will also be determined on the basis of the concentrations measured by means of the Ecological Risk Assessment method.

## Materials and methods

### The biocides which were studied

Table 2 provides the chemical structures and the physicochemical properties of the following compounds: diuron, isoproturon, cybutryne, and terbutryn, which are investigated in this study. Each of the analytical standards was purchased from Sigma-Aldrich (Germany). The analytical grade reagents, methanol, and ethyl acetate were purchased from POCH (Gliwice, Poland). The solutions of individual standards and their mixture were prepared in methanol stored in the dark at 4 °C. The concentrations of starting compounds were given in micrograms per milliliter: for isoproturon 13.5, diuron 35.5, terbutryn 5.5, and cybutryne 19.5, respectively. The deionized water (< 0.07 S/cm), used in order to perform solid phase extraction (SPE), was obtained from the HLP5 pure water system (Hydrolab, Gdańsk, Poland). The extraction cartridges for SPE: Oasis HLB (3 ml/60 mg) was purchased from Waters (Wexford, Ireland), and Chromabond DVB (3 ml/200 mg) was purchased from Macherey-Nagel (Düren, Germany).

**Table 2:**
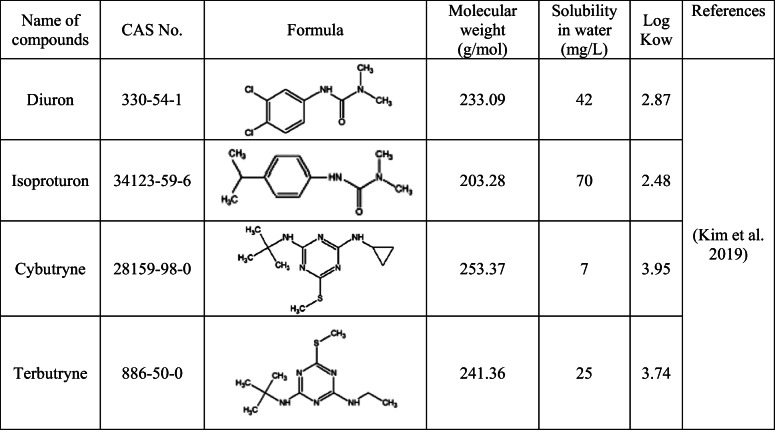
Biocides used in the study

### Sampling

**Fig. 1:**
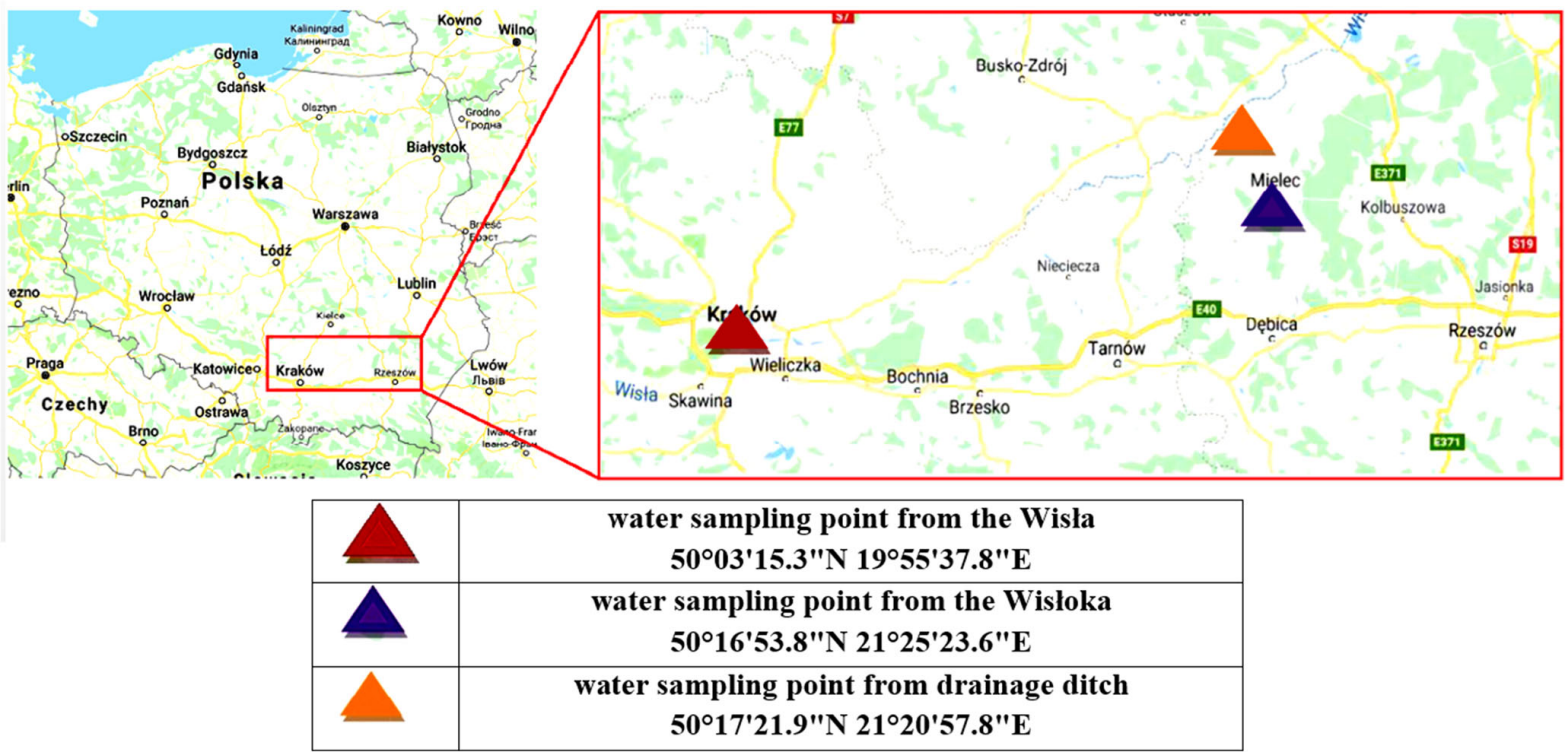
The location of the collection of the research samples (www.google.pl 2019)

The environmental water samples were taken from three locations, from the Wisła River in the Kraków section of the river course, from Wisłoka near Mielec, and from the water from the drainage located in the Borowa commune in the Mielec district. These places are shown on the map in Fig. 1. The Wisła is the longest Polish river, and Kraków is the largest city in its lower reaches. Distance from the sampling point on the Wisła (Kraków) to the mouth of the Wisła to the Baltic Sea is 865 km. The average flow rate of Wisła in Kraków was 98 m^3^/s (IMGW 2019a). This place was chosen for sampling, because the analyzed samples represent the great Polish metropolises and show the impact of urbanization on the presence of biocides in surface waters. According to the information provided by the Chief Inspectorate of Environmental Protection in Poland, the annual average electrolytic conductivity (EC) of the Wisła River in Kraków was 2996 μS/cm (Główny Inspektorat Środowiska 2018). The total dissolved solids (TSD) calculated by multiplying EC by a constant value of 0.64 characteristic for freshwater (Rusydi 2018) was 1917.44 mg/L. The Wisłoka is a river in the southeastern part of Poland; it is a right-bank tributary of the Wisła. Distance from the sampling point in Wisłoka (Mielec) to the mouth of the Wisłoka River to the Wisła is 22 km, but distance from the mouth of the Wisłoka to the Wisła to the mouth of the Wisła to the Baltic Sea is 713 km. The average flow rate of Wisłoka in Mielec was 28.7 m^3^/s (IMGW 2019a). The water samples were taken at Mielec, because the city is one of the main industrial centers in the Podkarpackie Province (the leading industries include aviation, the automotive, metalworking and plastics processing industries) and may be considered representative for small cities all over Poland. The annual average EC of Wisłoka was 398 μS/cm, and so, the calculated TSD value (Rusydi 2018) was 254.72 mg/L, so it can be expected that the pollution was much lower than in the Wisła (Główny Inspektorat Środowiska 2018). The drainage ditch collects rainwater from agricultural fields and nearby farms. It can be considered representative for Polish drainage channels located in villages. This choice will enable us to indicate the potential impact of agriculture on the presence of biocides in surface waters.

Three rounds of the investigation of similar samples were conducted. Two of them were carried out in the spring, in May and June 2018, and the third one in the winter, in February 2019. Three samples with a volume of 2.5 L each were taken to amber bottles from every place. The samples were taken in accordance with the ISO 5667-6:2016-12 standard. In the case of the Wisła and Wisłoka River at a depth of about 22–50 cm below the water table, while in the case of the drainage ditch, the sampling depth was about 15 cm, which was one-third of its depth (ISO International Organization for Standardization 2014). All samples were transported back to the laboratory in a dark and ice cool box and then vacuum filtered through glass-fiber filters purchased from Macherey-Nagel, firstly MN GF-1 (0.7 μm, 47 mm) and subsequently through MN GF-5 (0.4 μm, 47 mm).

### Solid-phase extraction

The recovery of biocides in the solid phase extraction process was determined. Two types of columns were used for the comparison of results: Oasis HLB cartridges (3 ml/60 mg) contain the Oasis HLB sorbent, which is a universal polymeric reversed-phase sorbent that was developed for the extraction of a wide range of acidic, basic, and neutral compounds from various matrices and EASY-polar modified polystyrene-divinylbenzene (3 ml/200 mg) known as Chromabond DVB. The test was triplicate for each kind of cartridges. Before use, the SPE cartridges were conditioned successively with 2 ml of methanol and 2 ml of deionized water. The samples were mounted on columns using the 12-position system for SPE by J.T. Baker (Netherlands) in a set with a diaphragm pump. After loading, the cartridges were dried under vacuum for 20 min. After elution with 4 ml of methanol, the collected extracts were evaporated to dryness with a gentle stream of nitrogen and redissolved in 0.5 ml of methanol. The solutions which were prepared were subsequently analyzed by gas chromatography. The recovery percentage, i.e., the accuracy, was calculated according to the formula:

$$ \frac{x_i}{\mu}\cdotp 100\%, $$

where *x*_*i*_—the quantity of analyte determined in the test sample;*μ*—the known amount of analyte in the test sample (International Organization for Standardization 2005).

The environmental samples after filtration were extracted by using Oasis HLB columns in the same way as described above and analyzed by CG-MS.

### Analytical procedure

Analyses were performed by means of a Thermo Scientific GC Trace 1300 gas chromatograph coupled with a ITQ 900 ion trap mass spectrometer and a TriPlus RSH autosampler. The components of the tested sample were separated using the Rxi®-5Sil MS capillary column from Restek (5% diphenyl, 95% dimethylsiloxane) with a length of 30 m, internal diameter of 25 m, and film thickness of 0.50 μm. The carrier gas was helium (99,999%) with a flow rate of 1 ml/min. The conditions for temperature separation were as follows: it began with a temperature of 70 °C maintained for 2 min, then there was a temperature increase, at a rate of 20 °C/min, to 320 °C, and it was maintained for 4 min. The programmable temperature of the vaporization injector was maintained at 250 °C, the transfer line at 250 °C, and the ion source at 250 °C. The injector was operated at splitless conditions for 2 min and then turned to the split mode at the ratio of 50:1. The analyses were performed in positive mode at an electron energy of 70 eV and an emission current of 250 μA. The volume of injections was 1 μL. The mass spectrometry analyses were performed in the multiple reaction monitoring (MRM) mode, measuring the fragmentation of the precursor ions. The choice of fragmentation products for each substance was based on the strongest signal. The presence of biocides in individual samples was tested on the basis of ions. Table 3 lists individual compounds with the corresponding ions and retention times. The precursor ion was bolded. The obtained results were processed using the specialized Xcalibur® software.

**Table 3:** Ions and retention time for individual biocides and their calibration data

Biocide	Retention time (minute)	Precursor–products ions, *m*/*z*	*R*^2^	Concentration range of the calibration solutions (ng/L)	Method detection limit (MDL) (ng/L)
Isoproturon	9.93	*178*–146, 128	0.9987	3–2242	0.9
Diuron	10.07	*219*–187, 174	0.9958	8–5921	2.5
Terbutryn	12.01	*185*–170, 157	0.9854	1.5–917	0.4
Cybutryne	12.77	*182*–109, 140	0.9992	4–3246	1.4

Italicized text means the precursor ion

The calibration curves were prepared on the basis of eight solutions. On the basis of the latter, the validation parameters were determined: detection limit (LOQ), detection limit (LOD), and linearity of calibration lines. The results which were obtained are presented in Table 3. All four calibration curves are characterized by a very high correlation coefficient *R*^2^ of 0.9987–0.9854, which in practice means that the linear nature of the relationship between the concentration of a given biocide and the size of the peak obtained after the analysis is very likely. The linearity of the analytical method is to show that the obtained measurement results are directly proportional to the concentration (content) of the substance determined in the sample, within a given range. The condition of linearity is that the correlation coefficient *R* exceeds 0.98 (the case of detection of residual substances) (International Organization for Standardization 2005). Owing to this relationship, the concentrations of biocides in environmental samples were calculated.

### ERA

In accordance with the guidelines described in the European Union documents, an Ecological Risk Assessment (ERA) was performed to determine the probability of negative effects, caused by the presence of different compounds in the environment so show the toxicological risk level. The ERA procedure described in the European Chemicals Agency (ECHA) as well as the Technical Guidance Document on Risk Assessment (TGD) is based on the calculation of the risk factor (RQ) according to the formula:

$$ \mathrm{RQ}=\frac{\mathrm{MEC}}{\mathrm{PNEC}} $$

where the MEC value is the measured concentration level of a pollutant in the environment, while the PNEC is the value of the predicted no effect concentrations for the structure and the functioning of the ecosystem (ECHA 2017). It means that the PNEC concentration for an environmental component is the concentration below which there is no negative effect on ecosystems. The PNEC value is obtained from the available information on species toxicity in the relevant environment as well as from the toxicity endpoints (LC50—the concentration lethal to 50% of organism or NOEC—the concentration effect no observed), using the appropriate assessment factors (European Commission 2003; ECHA 2017). The legislator specifies that if the RQ value is equal to or above 1, it is considered that the potential risk to the environment is likely to occur; otherwise, there is no such risk.

### Statistical analysis

The analyses were performed by means of Statistica (version 13.0). Their purpose was to check whether both the differences between the tested column fillings and the differences between the results of analyses of environmental samples in the spring (May and June) differ significantly in terms of statistics. The hypotheses were tested by means of the Student *t* test. The statistical level of significance was set as *p* < 0.05.

## Results and discussion

### The efficiency of the SPE

**Fig. 2:**
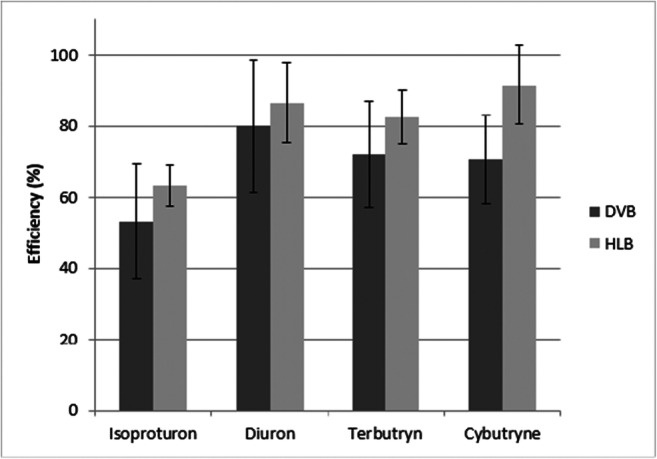
The efficiency of the SPE. Bars represent standard deviations between samples

The collected results of the SPE process performance for two columns, HLB Oasis and Chromabond DVB, are shown in Fig. 2. The bars show the average recovery for individual compounds. Error bars in the form of standard deviation were also applied.

After carrying out the Student *t* test for the significance level *p* = 0.05, the results of recoveries for both columns do not show statistical differences. This means that in statistical terms, the observed results are equal. However, it was decided that in the research in the detection of biocides in environmental waters, the second type of the tested columns, designated as HLB and containing the Oasis HLB sorbent, should be selected. The first column, marked as DVB (EASY-polar modified polystyrene-divinylbenzene), demonstrates a lower efficiency of the SPE process. The disadvantage of this type of column filling is the lower repeatability of results, as evidenced by the higher values of the standard deviation. For the analyzed biocides, recoveries were higher for the HLB columns than for the DVB columns by 19% for isoproturon, 8% for diuron, 14% terbutryn, and 29% cybutryne. In connection with the above, for the further analyses on the environmental samples, the columns that dropped in the tests better were used, i.e., the HLB columns. Moreover, the fact that there are reports in the literature on the use of these cartridges for analyzing the selected biocides in water suggests the choice of Oasis columns (Agüera et al. 2000; Dolores Hernando et al. 2001; Carabias-Martínez et al. 2004; Mazzella et al. 2010; Robles-Molina et al. 2014).

### Concentrations of biocides

#### Concentrations in environmental samples

The content of biocides in environmental samples was determined on the basis of water samples taken and the execution of the ten replicates of the study. Table 4 presents the final results for individual sampling sites in the form of an average result with a standard deviation. The coefficient of variation ranges from 2.17 to 11.23%. The tests that were performed can be considered as done correctly because the coefficient of variation for the results of the determination of the ingredients content at the trace level should not be greater than 15% (International Organization for Standardization 2005).

**Table 4:** Results of the study of the biocide content in the environmental samples

		The Wisła River	The Wisłoka River	The drainage ditch
		Average concentration (ng/L)	Average concentration (ng/L)	Average concentration (ng/L)
Isoproturon	May	53.24 ± 1.48	35.92 ± 2.75	30.87 ± 1.13
	June	55.20 ± 1.20	34.62 ± 3.43	29.67 ± 2.93
	February	< MQL	< MQL	< MQL
Diuron	May	77.71 ± 1.30	51.58 ± 2.54	72.01 ± 2.61
	June	64.80 ± 3.57	48.40 ± 3.05	69.09 ± 7.76
	February	< MQL	< MQL	< MQL
Terbutryn	May	9.49 ± 0.76	1.94 ± 0.04	6.60 ± 0.18
	June	12.02 ± 0.39	2.66 ± 0.26	7.07 ± 0.52
	February	< MQL	< MQL	< MQL
Cybutryne	May	19.92 ± 0.69	14.72 ± 1.09	9.52 ± 0.47
	June	20.97 ± 1.23	15.07 ± 0.80	10.44 ± 1.00
	February	< MQL	< MQL	< MQL

None of the selected biocidal products was determined in the winter samples. In this case, the results of all analyses were below MQL, while biocides were found in all spring samples. Bacigalupo and Meroni also observed similar seasonal relationships in their studies. In the water samples collected once a month from an agricultural area in the north of Italy, they analyzed, inter alia, diuron. These studies demonstrated no diuron in an irrigation ditch in the winter months, i.e., from October to February inclusive (Bacigalupo and Meroni 2007). Also, in northern Italy during the winter from February to April, concentrations of various compounds, including some biocides, were analyzed in river water. Isoproturon concentrations were very low, ranging from undetectable to 0.5 ng/L, which is fully compatible with this research, while diuron concentrations ranged from undetected to 46 μg/L (Loos et al. 2007). Studies conducted in France in various river waters of the Brittany region show that biocide pollutions in winter are episodic. Their concentrations in December ranged from not determined over LOQ to 522 ng/L and 42 ng/L for isoproturon and diuron, respectively (Gervais et al. 2008).

The biocide concentrations in the spring trials were as described below. After statistical analysis, it was found that as far as isoproturon and cybutryne are concerned, in each of the sampling points, there is no significant difference in the results obtained for the May and June water analyses. This means that the amount of compounds emitted during the period considered can be regarded as being at the same level. For diuron, the only significant differences were noted in the water from the Wisła River.

The highest concentrations of isoproturon were recorded in the samples taken from the Wisła River in Kraków—it was over 50 ng/L, whereas the concentration in the samples from the Wisłoka River and the drainage ditch were lower by about 40—they were 35 and 30 ng/L, respectively. The obtained concentrations are in good agreement with the results of tests carried out in Greece and Poland. In the north of Greece, the waters of Strymonas and Nestos were tested. These analyses demonstrated the presence of isoproturon in both rivers with the following concentrations: Strymonas 10 ng/L and Nestos 339 ng/L (Papadakis et al. 2018). Isoproturon was detected in almost one-third of surface water analyses carried out in the Wielkopolska Province (Poland). Its concentrations were between 10 and 60 ng/L (Drożdżyński et al. 2009).

In the case of diuron, the lowest concentrations of this biocide were detected in a sample taken from the Wisłoka and it was 50 ng/L. However, samples taken from the Wisła and drainage ditch contained similar amounts of diuron, the concentration of which was detected at roughly 70 ng/L. The concentrations obtained are similar to the results of an analysis carried out in Italy in 2006 in agricultural areas. In the ditch water samples in May, the concentration was 60 ng/L and in June 73 ng/L (Bacigalupo and Meroni 2007). Compared with research conducted in recent years, the concentrations obtained are higher than those recorded in Italy and Greece. In the Gulf of Naples, diuron concentration ranged from 1.6 to 34.8 ng/L, and in the Gulf of La Spezia, it was in the range of 1.0–28.2 ng/L (Ansanelli et al. 2017), while in Greece in Styminas, it was below LOQ (Papadakis et al. 2018). In Japan in the years 2014–2015, research was conducted on the impact of agriculture and urban activities on biocide pollution in the Kurose River (Higashi Hiroshima). In the bodies of water which were analyzed, diuron was identified at all points from which samples were taken, and its maximum concentration was at the level of 4620 ng/L, which is almost 70 times higher than that obtained in our studies. The presence of diuron proved to be closely related to human agricultural activity, which was confirmed by the fact that the diuron concentration was connected with the data associated with the use of biocidal compounds in the Hiroshima Prefecture (Kaonga et al. 2015).

The water collected from the Wisła turned out to be the most contaminated also with terbutryn (approximately 10 ng/L), whereas the water collected from the Wisłoka manifested its lowest amount, i.e., five times less. This chemical compound also occurs in the waters of the drainage canal at slightly lower quantities than in the Wisła. In Denmark’s surface waters, the highest terbutryn concentration that was determined was 14 ng/L in the water from a lake near Slagelse, while in Hungary, the maximum contaminant level in water was 2 ng/L (Maloschik et al. 2007; Vorkamp et al. 2014). The results of our analysis are comparable with those mentioned above. The presence of terbutryn was also examined in the waters of the Vltava River in the Czech Republic and the waters of the Spanish river Ebro. The concentrations reported in the literature were about 50 times higher than those obtained as a result of our analyses. Terbutryn was found in Vltava at the level of 200 ng/L, in Ebro—500 ng/L (Barceló et al. 1995; Ormad et al. 2008). In contrast, in Germany, surface water pollution with terbutryn was even higher; the highest value which was recorded on the Weschnitz river was as much as 5600 ng/L. The source of this biocide is considered to be agriculture, or rather, the pesticides used in it (Quednow and Püttmann 2007).

In the case of cybutryne, the highest concentrations were recorded in the samples taken in Kraków from the Wisła—20 ng/L; lower concentrations of biocide were detected in the Wisłoka at a level of 15 ng/L, and the lowest in the water collected from the Mielec drainage ditch—10 ng/L. No concentration was above maximum allowable concentrations environmental quality standards (MAC)-EQS and annual average AA-EQS, except cybutryne, where (MAC)-EQS and AA-EQS were exceeded by samples from Kraków and all samples, respectively (see Table 1). Cybutryne concentrations in surface waters throughout the whole wall range from a few to several dozen nanograms per litre. In the Danish port of Copenhagen, its concentration was 13 ng/L and it was released into the water from mooring ships (Ormad et al. 2008). In Great Britain, cybutryne with concentrations ranging from a few to several dozen nanograms per litre was found in several rivers (Gatidou et al. 2007). Cybutryne was analyzed in waters taken from the Bay of Naples (0.8 to 135.5 ng/L) and from the Bay of La Spezia (from 0.2 to 9.7 ng/L). In both of these cases, diuron and cybutryne concentrations in the north of the country were much lower than in the south. Moreover, it was mainly observed in the Gulf of Naples that the level of biocides increased from winter to summer, which is due to the sailing season (Ansanelli et al. 2017). In the waters of the Kurose River in Japan, cybutryne was only found in a sample from Izumi, a dense urban and industrial area, and biocide paints that were used to paint the façades of buildings were recognized as its source (Kaonga et al. 2015).

Available publications suggest that precipitation has an impact on the concentration levels in surface waters, as the load of a given compound increases as a result of surface runoff (Konstantinou and Albanis 2004; Wittmer et al. 2010; Kaonga et al. 2015; Ansanelli et al. 2017). According to the Bulletin of the National Hydrological and Meteorological Service for the meteorological station in Kraków (reference station for the Małopolska province), as well as for station in Rzeszow (reference station for the Podkarpackie province), May 2018 was considered as normal, while June as too dry, according to the Z. Kaczorowska classification (IMGW 2018). The classification allows to estimate the shortage or excess of precipitation in relation to the long-term (1971–2000) standard. The total precipitation in May and June for Kraków was 59 and 39 mm, while for Mielec, it was 42 and 24 mm, respectively. The month of February 2019 was included in the Bulletin of the National Hydrological and Meteorological Service for the meteorological stations in Kraków and Rzeszow (IMGW 2019b) as an extremely dry period, which was based on the same classification. The total precipitation in February for Kraków was 9 mm, while for Mielec, it was 6 mm. Taking into account the literature reports, it is believed that rain of at least a dozen or so mm in day may cause an increase in the concentrations of selected substances in surface watercourses (Wittmer et al. 2010). Due to the low rainfall, in sampling periods, it is difficult to find a connection between the occurrence of precipitation and the concentration of biocides in surface watercourses; however, we do not completely rule out the impact of such dependence.

These biocides are widely used in agriculture, industry, and individual households because the former act as biocidal and especially as algicidal, herbicidal agents. Their presence in surface waters in all three locations confirms the use of biocides both in agricultural areas, which correspond to samples taken from the drainage ditch, as well as in urban areas such as Mielec, or even in large agglomerations such as Kraków. The research that was conducted does not provide an answer to the question about the origin of biocides in a given location. The obtained concentration values are similar, which demonstrate an equally high pollution of surface waters regardless of the place of sampling. It is believed, however, that in an area where agriculture predominates, biocides can first of all get into surface waters as a result of direct runoff from arable fields, or when washing equipment is used to distribute these materials. The above statement confirms the fact that biocides were detected only in samples taken in spring, i.e., during the period of agricultural activity, and that cybutryne, which is a commonly used herbicide, had the highest concentration in a drainage ditch located in rural areas. In cities, surface runoff is important as biocides used in anti-fouling agents get into watercourses with it. The said substances, among other things, prevent the appearance of microorganisms on the façades of buildings, or are used as wood preservatives (Styszko et al. 2015; Styszko and Kupiec 2018). Another source of biocides is runoff from non-agricultural areas whereunnecessary vegetation is destroyed. They are also delivered to surface water along with the discharge of sewage treated. In Kraków, small tourist ships are seen along the Wisła River in the summer, from May to September. It is believed that the highest diuron concentration in the Wisła River in the Kraków section is caused by the use of impregnating agents for the maintenance of ship hulls. This is confirmed by the fact that during the winter, no biocides were determined in the water samples from the Wisła River, compared with the samples taken at the turn of May and June, which coincides with the start of the tourist season for tourist ships.

#### Concentrations in relation to directive 2013/39/EU

Environmental samples were collected three times, so the biocide content cannot be validated according to directive 2013/39/EU in terms of the annual intake but only as the permissible maximum (Table 1). Three of the biocides under consideration, i.e., isoproturon, diuron, and terbutryn, manifest concentrations below the maximum allowable concentration in the tested environmental samples. Cybutryne, as far as the samples taken from the Wisła River are concerned, is below the maximum annual concentration. The permissible concentration was exceeded by 16%. In the case of research conducted in the water from the Wisłoka River, the concentration of this biocide is on the verge of exceeding the maximum permissible concentration, while as far as the drainage ditch is concerned, this value has been exceeded by 25%.

#### Concentrations in relation to environmental inspectorate in Poland

These biocides which are discussed should be monitored in surface waters as priority substances in accordance with the European guidelines (The European Parlament and the Council of the European Union 2013). GIOŚ (pl. The Chief Inspectorate for Environmental Protection) carries out an assessment of the state of rivers and dam reservoirs. The sampling points for this study were located on the Wisła River between the GIOŚ monitoring points of Wisła-Kopanka (before the point of sample taking) and Wisła-Grabie (after the point of sample taking), while on the Wisłoka—between Wisłoka-Rzochów (before the point of sample taking) and Wisłoka-Gawłuszowice (after the point of sample taking). The GIOŚ report from 2017 to 2018 contains only concentrations for two of the four biocides discussed, namely diuron and isoproturon, which were determined in 2017 for the monitoring points of Wisłoka-Rzochów and Wisłoka-Gawłuszowice. The maximum concentrations were below the limit of quantification (LOQ = 100 μg/L) (Główny Inspektorat Środowiska 2018).

### Risk estimation

The risk quotations (RQ) value was calculated for the measured concentration of each biocide in the environmental samples taken in spring using the conventional method for environmental risk assessment (ERA). The predicted no-effect concentrations (PNEC) values for individual biocides tested were reported by Tousova et al. (Tousova et al. 2017). The authors applied newly developed simplified Effect-directed analysis (EDA) protocol within a European demonstration program (EDP), which included effect-based monitoring of contaminants of emerging concerns at 18 sampling sites in 4 European river basins. The main objectives of the study were to link biological effects to target compounds and to estimate their risk to aquatic biota. Herbicides, with minor contribution from other micropollutants, were linked to the observed toxicity to algae. Twenty-one target compounds, among other, terbutryn, were prioritized on the basis of their frequency and extent of exceedance of predicted no effect concentrations. The results of this work highlighted that some priority compounds are still relevant for monitoring in the EU, even though several of them were phased out for major application, e.g., terbutryn, what is in agreement with our study. Compound concentrations lower than PNECs are considered safe, while concentrations exceeding PNECs are likely to cause unacceptable (i.e., hazardous) effects on aquatic organisms. The estimated risk values for the analyzed compounds are presented in Table 5—which does not include the results from the winter campaign, due to the fact that the biocide’s concentration levels were < MQL. The RQ value is below 1 for each samples collected in February, suggesting that in winter, no pose risk was associated with the environmental presence of the analyzed biocides.

**Table 5:** Environmental risk values calculated by means of the environmental risk assessment method with reference to the PNEC values provided in the literature for the studied areas (Tousova et al. 2017)

		Isoproturon		Diuron		Terbutryn		Cybutryne	
		PNEC (ng/L)	RQ	PNEC (ng/L)	RQ	PNEC (ng/L)	RQ	PNEC (ng/L)	RQ
The Wisła River	May		0.18		0.39		1.46		7.97
	June		0.18		0.32		1.85		8.39
The Wisłoka River	May	300	0.12	200	0.26	6.5	0.30	2.5	5.89
	June		0.12		0.24		0.41		6.03
The drainage ditch	May		0.10		0.36		1.02		3.81
	June		0.10		0.35		1.09		4.18

The risk quotient values of all the discussed results from the spring campaign vary from 0.1 to 8.39. The RQ values for isoproturon and diuron for each of the samples and for terbutryn in the sample from the Wisłoka River are below 1. According to the European guidelines (European Commission 2003), these compounds do not pose risk for the aquatic environment. In the studies of Tsaboula et al. (2016), isoproturon was also considered to be of low risk for the aquatic environment, while diuron has been classified to category of compounds suspected of having adverse impact on human health and are less likely to pose a significant long- or short-term risk to the aquatic environment. According to Tousova et al. (2017), diuron among others herbicides were found to be one of the dominant components causing algal growth inhibition at 14 out of 18 sampling sites. The concentration of diuron at 150 ng/L was sufficient to explain the observed effect independently of the other compounds detected in the tested extracts. In the Wisła River and the drainage ditch, the amount of terbutryn is alarming. The RQ values of this compound in these places are between 1.02 and 1.85, which indicates a risk of harmful health effects. Terbutryn as a herbicide shows toxicity to aquatic organisms, especially microalgae, where in order to induce a 50% reduction in growth, it is enough to expose 72 h to the concentration of 2 μg/L (Okamura et al. 2000). In all analyzed samples in reference to cybutryne, regardless of location and the period of collecting the sample (spring), obtained RQ values are definitely above 1. This indicates that the potential risk of a negative impact on the aquatic ecosystem occurred in every analyzed research point. The highest RQ was detected in the drainage ditch (about 8). It might cause the process of photosynthesis to slow down or even stop, and this in turn may lead to the occurrence of toxic effects on higher plants and many aquatic organisms, including phytoplankton (Ur Rehman et al. 2019). In addition, cybutryne has a low PNEC value, which is reflected by a high RQ, and thus, it can potentially cause ecological effects.

In the case of diuron, the obtained RQ values are in good agreement with those given in the specialist literature. In publications concerning European rivers, the RQ values observed for diuron was below or equal to 1 (Deng et al. 2012; Palma et al. 2014; Tsaboula et al. 2016; Tousova et al. 2017; Carazo-Rojas et al. 2018; Shao et al. 2019) which means no adverse effects due to the presence of this biocide in water. A similar situation occurs in reference to terbutryn—the RQ value obtained in this work is reflected in other authors’ research The RQs have been found in the literature as no more than 0.1 (Tsaboula et al. 2016; Tousova et al. 2017; Gallé et al. 2019). The calculated RQ value for isoproturon is slightly above 0.1, so it only a little higher than the value provided in the available literature, which is below 0.1 (Palma et al. 2014; Ccanccapa et al. 2016; Tousova et al. 2017). The difference is in the order of hundredths. In the case of cybutryne, the scientific literature which was analyzed indicates that the highest RQ in river waters was 0.435 (Gallé et al. 2019) and < 0.1 (Tousova et al. 2017). These values are almost 10–20 times lower than the calculated RQ. According to research by Nyström et al. concerning freshwater, inhibition of photosynthesis for phytoplankton occurs when RQ > 1.2 (Nyström et al. 2002). It means that in the drainage ditch, the process of producing organic compounds may be disturbed, and in the Wisła and Wisłoka River even significantly limited. Reducing photosynthesis in the phytoplankton might negatively affect the higher order organisms that depend on it.

The logarithm of the octanol-water partition coefficient (Log Kow) is inversely proportional to the ability of the compound to adsorb to soil or sediments (Kaonga et al. 2015). According to the log Kow values ​presented in Table 2, the adsorption capacity of the compounds in question is as follows: cybutryne > terbutryn > diuron > isoproturon(Kim et al. 2019). This means that cybutryne shows a much lower ability to transfer to surface water reservoirs, while isoproturon is eluted best. The above dependence is also confirmed by the solubility in water, which is the highest for isoproturon and the lowest for cybutryne (Table 2). Research by Albanis et al. and Wittner et al. indicate that there is a phenomenon of cybutryne retention on the soil, as a result of which it is possible to wash it out by precipitation even a month after its application (Albanis et al. 2002; Wittmer et al. 2010). Cybutryne in freshwater has a half-life of 200 days (Fink and Slothuus 2013), and in salt water, it can reach up to 350 days (Fernandez and Gardinali 2016), so it is difficult to degrade. The discussed biocide poses a threat to the environment through the possibility of accumulation in sediments and plants (Nyström et al. 2002). The bioaccumulation of cybutryne in fish is moderate (bioconcentration factor 250 L/kg), but the compound is rapidly eliminated, even in less than 3 days (Fink and Slothuus 2013). Cybutryne is present in sediment and plankton at levels 0.01–0.09 and 0.075–0.45 μg/g dry weight, respectively (Balakrishnan et al. 2012). The log Kow for terbutryn is 3.74 (Kim et al. 2019). It is a highly lipophilic compound, which indicates its bioaccumulation (Brandhorst Daho 1994). Terbutryn is resistant to hydrolysis and is present in environmental sediments (Masiá et al. 2015). Its half-life is higher than cybutryne and amounts to 177–644 days (Arufe et al. 2004). Isoproturon shows the highest water solubility among the analyzed biocides (Kim et al. 2019). It is not as easily sorbed as cybutryne, so it is easily leachable into water (Masiá et al. 2015). Isoproturon half-life is the shortest and in water is 30 days and in soils 40 days (World Health Organization (WHO) 2003). The general tendency shows higher concentrations of biocides in freshwaters compared with salt waters (Daniels and Layton 1988; Donald et al. 1995; Vorkamp et al. 2014). The average annual salinity of the Wisła is 7.5 times higher than the Wisłoka’s (Główny Inspektorat Środowiska 2018). Hence, authors expected higher concentrations of biocides in the Wisła River. It is assumed that as the salinity of a given water increases, the amount of pollutants increases. The greater the TSD is, the more contaminants can be found in the water. The conducted research confirms the above thesis, because for each of the analyzed compounds, the concentration of biocides in the Wisła was higher than in the Wisłoka—there were 1.5 times more isoproturon and diuron, 4.5 times more terbutryn, and 1.3 times more cybutryne.

Taking into account all the above information about the harmfulness of the analyzed biocides in relation to individual elements of the environment, it is considered that the list of priority substances in the field of water policy has rightly been extended with selected biocides. Toxicity, long half-life, as well as the bioaccumulation capacity of biocides are the factors that cause the greatest risk to living organisms.

## Conclusions

The aim of this study is to respond to trends observable in the modern world, namely to increase interest in environmental issues in the context of the continuous industrialization and development of agriculture. The analyses of environmental samples clearly showed that the biocidal substance abundance in surface waters is seasonal. The quality of the waters tested was determined on the basis of directive 2013/39/EU. The current biocide concentration standards in waters were exceeded only in the case of cybutryne samples from the Wisła River, and in the case of the Wisłoka River, they almost exceed the permissible threshold. The most polluted sample turned out to be the water from the Wisła River. It should be emphasized that biocides in the aquatic environment are present in such high concentrations that, on the basis of ERA calculations, they can pose a potential threat to the aquatic environment and cause chronic effects.

## Data Availability

All data generated or analyzed during this study are included in this published article.
